# The x_c_^−^ cystine/glutamate antiporter: a mediator of pancreatic cancer growth with a role in drug resistance

**DOI:** 10.1038/sj.bjc.6604485

**Published:** 2008-07-22

**Authors:** M Lo, V Ling, Y Z Wang, P W Gout

**Affiliations:** 1 Department of Experimental Medicine, University of British Columbia, Vancouver, BC, Canada V6T 2B5; 2 Department of Cancer Genetics, British Columbia Cancer Agency, Vancouver, BC, Canada V5Z 1L3; 3 Department of Cancer Endocrinology, British Columbia Cancer Agency, Vancouver, BC, Canada V5Z 1L3; 4 The Prostate Centre at Vancouver General Hospital, Vancouver, BC, Canada V6H 3Z6

**Keywords:** pancreatic cancer, cystine transporter, glutathione, drug resistance, xCT

## Abstract

The x_c_^−^ cystine transporter enhances biosynthesis of glutathione, a tripeptide thiol important in drug resistance and cellular defense against oxidative stress, by enabling cellular uptake of cystine, a rate-limiting precursor. Because it is known to regulate glutathione levels and growth of various cancer cell types, and is expressed in the pancreas, we postulate that it is involved in growth and drug resistance of pancreatic cancer. To examine this, we characterised expression of the x_c_^−^ transporter in pancreatic cancer cell lines, MIA PaCa-2, PANC-1 and BxPC-3, as subjected to cystine-depletion and oxidative stress. The results indicate that these cell lines depend on x_c_^−^-mediated cystine uptake for growth, as well as survival in oxidative stress conditions, and can modulate x_c_^−^ expression to accommodate growth needs. Immunohistochemical analysis showed that the transporter was differentially expressed in normal pancreatic tissues and overexpressed in pancreatic cancer tissues from two patients. Furthermore, gemcitabine resistance of cells was associated with elevated x_c_^−^ expression and specific x_c_^−^ inhibition by monosodium glutamate led to growth arrest. The results suggest that the x_c_^−^ transporter by enhancing glutathione biosynthesis plays a major role in pancreatic cancer growth, therapy resistance and represents a potential therapeutic target for the disease.

Pancreatic cancer is the second most common gastrointestinal malignancy and the fourth leading cause of cancer-related deaths in the United States (Freelove and Walling, 2006). It is highly resistant to conventional chemotherapeutic regimens (Zalatnai and Molnar, 2007) and gemcitabine (GEM), currently the standard chemotherapeutic agent for treatment of pancreatic cancer, is only marginally effective (Wolff, 2007). Clearly, there is a critical need for establishing new targets and approaches for therapy of pancreatic cancer.

Glutathione (GSH) is a tripeptide thiol consisting of glutamate, cysteine and glycine, which plays a critical role in cellular defenses against oxidative stress and toxic compounds (Griffith, 1999). In cancer cells the GSH levels maintain DNA synthesis, growth and multidrug/radiation resistance, and sustenance of GSH levels through GSH biosynthesis is vital for growth and survival of tumours. As such, GSH is considered an important target in cancer therapy and various therapeutic approaches based on GSH depletion of cancer cells have been suggested (Schnelldorfer *et al*, 2000; Estrela *et al*, 2006; Doxsee *et al*, 2007). Glutathione biosynthesis is critically dependent on availability of intracellular cysteine, a major rate-limiting factor (Griffith, 1999). Although tissues such as the liver can synthesise cysteine from L-methionine through the transsulphuration pathway (Rosado *et al*, 2007), certain experimental cancers (e.g., lymphomas, gliomas) are incapable of synthesising adequate amounts of the amino acid for GSH synthesis and hence depend for growth and viability on uptake of extracellular cysteine or cystine, the oxidised form. In such cases, reduced cellular uptake of the amino acid can lead to depletion of intracellular GSH levels and subsequent growth arrest (Gout *et al*, 1997; Iwata *et al*, 1997; Chung *et al*, 2005).

The x_c_^−^ cystine/glutamate antiporter is a plasma membrane transporter mediating cellular uptake of cystine in exchange for intracellular glutamate with a stoichiometry of 1 : 1 (Lo *et al*, 2008). It is a member of a family of heterodimeric amino-acid transporters, plasma membrane proteins composed of a heavy subunit and a light subunit coupled through a disulphide bridge. The human x_c_^−^ transporter consists of the heavy subunit 4F2hc, found in a variety of amino-acid transporters, coupled to the light subunit, xCT, which confers specificity for cystine (Verrey *et al*, 2004). It has been demonstrated, both *in vitro* and *in vivo* that inhibition of the x_c_^−^ transporter in cancer cells depending on extracellular cystine/cysteine for growth and viability, can lead to reduced uptake of this amino acid with subsequent depletion of intracellular GSH levels and growth arrest (Gout *et al*, 2001; Chung *et al*, 2005; Doxsee *et al*, 2007). For such malignancies the x_c_^−^ transporter therefore represents a potential therapeutic target (Lo *et al*, 2008).

Human xCT is expressed in the pancreas, as one of a limited number of tissues (Bassi *et al*, 2001). Although x_c_^−^ activity has been investigated in normal pancreatic cell lines (Sato *et al*, 1998), the function of the transporter in pancreatic cancer cells has so far not attracted much attention. In the present study, we have investigated a potential role for the x_c_^−^ transporter in growth, viability and GSH-based drug resistance of pancreatic cancer, using human pancreatic cancer cell lines, MIA PaCa-2, PANC-1, and BxPC-3, in particular as modulated by oxidative stress factors, changes in extracellular cystine levels and specific x_c_^−^ inhibition. Importantly, pancreatic cancer tissues from patients showed overexpression of the transporter. Taken together, the results suggest that the x_c_^−^ cystine transporter plays a significant role in the growth, viability and therapy resistance of pancreatic cancers and may be useful as a potential target for therapy of the disease.

## Materials and methods

### Materials

Chemicals, dyes, solvents, solutions and culture medium were obtained from Sigma-Aldrich Canada Ltd, Oakville, ON, Canada, unless otherwise indicated.

### Maintenance of pancreatic cancer cell cultures

The human pancreatic cancer cell lines, MIA PaCa-2, PANC-1 and BxPC-3, were originally obtained from the American Type Culture Collection (Manassas, VA, USA) and maintained as monolayer cultures in T75 tissue culture flasks (Nunc™, Roskilde, Denmark) in MEM containing 0.1 mM cystine (StemCell Technologies, Vancouver, BC, Canada), supplemented with 10% fetal bovine serum (FBS; Invitrogen, Carlsbad, CA, USA) and 3.6 g l^−1^ glucose at 37°C in a humidified chamber in an atmosphere of 95% air and 5% CO_2_.

### Assessment of growth requirements for exogenous cystine

Cells were plated in 96-well plates (Nunc™) at 1000 cells per well (in triplicate) in their maintenance medium overnight. Cells were then washed and overlaid with cystine- and methionine-deficient RPMI-1640 medium supplemented with 10% FBS and 2 mM glutamine, in the presence or absence of cystine (0.1 mM), methionine (0.1 mM), and/or cystathionine (0.15 mM) in various combinations. Following 72 h of incubation, a neutral red uptake assay was performed to determine cell survival. It is noteworthy that normal cystine concentrations in human blood plasma range between 0.1–0.2 mM (Chawla *et al*, 1984; Gmunder *et al*, 1991). Therefore, we defined the normal cystine concentration to be 0.1 mM cystine. Accordingly, cystine concentrations lower than 0.1 mM or higher than 0.2 mM were considered as low or high cystine concentrations, respectively.

### Cell survival/proliferation assay

Cell proliferation and viability were determined by neutral red uptake (Babich and Borenfreund, 1991). Cells were plated in 96-well plates at 1000 cells per well. The next day, cells were treated as dictated by the particular experiments. Wells were then aspirated and incubated with 100 *μ*l 0.0025% neutral red dye in cell culture medium. Empty wells were also incubated with neutral red dye to allow for background absorbance correction. After 4 h of incubation, wells were aspirated and 100 *μ*l 1% acetic acid in 50% ethanol was added per well to solubilise intracellular neutral red dye. Absorbance was determined at 550 nm using a 96-well VERSA max microplate reader (Molecular Devices, Sunnyvale, CA, USA).

### RNA isolation and q-RT–PCR

Isolation of total RNA from cultured cells *in vitro* or tumour cells *in vivo* was performed using an RNeasy Micro kit (Qiagen Inc., Valencia, CA, USA) according to the manufacturer's recommendations. RNA (5 *μ*g) was used to synthesise first-strand cDNA using the Superscript II RT–PCR kit (Invitrogen), according to the manufacturer's recommendations. q-RT–PCRs were performed in 384-well plates using SYBR® Green PCR Master Mix (Applied Biosystems, Foster City, CA, USA) in a volume of 15 *μ*l (containing 50 ng of cDNA). Fluorescence emission was detected for each PCR cycle on a ABI Prism® 7900 Sequence Detection System (Applied Biosystems) and threshold cycle (*C*_t_) values were determined based on a 40-cycle reaction. Thermal cycling conditions were 50°C for 2 min and 95°C for 5 min, followed by 40 cycles of 15 s at 95°C, 30 s at 58°C, and 30 s at 72°C. Average *C*_t_ values from duplicate PCRs were normalised to average *C*_t_ values for the housekeeping gene *β*-2-microglobulin (*β*2M) from the same cDNA preparations. The relative mRNA expression of a gene was calculated as follows: Average *C*_t_ (*β*2M)−Average *C*_t_ (gene)=d; mRNA expression relative to *β*2M=2^(d). Each q-RT–PCR was carried out in duplicate.

Primer sequences are as follows: mouse 4F2hc: forward 5′-GAGGACAGGCTTTTGATTGCAG-3′, reverse 5′-AGGTAGGAGCTGGTCAACAGCA-3′; mouse xCT: forward 5′-GAGGACAGGCTTTTGATTGCAG-3′, reverse 5′-AGGTAGGAGCTGGTCAACAGCA-3′; mouse *β*2M: forward 5′-CACCCCCACTGAGAGACTGATACA-3′, reverse 5′-TGATGCTTGATCACATGTCTCG-3′; human 4F2hc: forward 5′-AGTGCCAACATGACTGTGAAG-3′, reverse 5′-CCTTACTCCGCTGGTCACTCAG-3′; human xCT: forward 5′-TGCTGGCTGGTTTTACCTCAAC-3′, reverse 5′-CCAATGGTGACAATGGCCAT-3′; human *β*2M: forward 5′-ACCATGTGACTTTGTCACAGCC-3′, reverse 5′-AATCCAAATGCGGCATCTTC-3′.

### Glutathione assay

Total GSH levels were measured using the ApoGSH™ GSH Colorimetric Detection Kit (BioVision, Mountain View, CA, USA) according to the manufacturer's recommendations.

### Western blotting

Total cellular protein was extracted on ice by sonication and supplemented with a protease inhibitor solution (one tablet of Complete Protease Inhibitor Cocktail dissolved in 25 ml of 1 × PBS) (Roche, Basel, Switzerland).

Protein samples (10 *μ*g) were electrophoresed under reducing conditions on NuPAGE 4–12% bis-Tris gels and transferred to 0.45-*μ*m nitrocellulose membranes (Invitrogen). Membranes were blocked overnight at 4°C with 10% skim milk in 1 × PBS, followed by incubation with the following primary antibodies: mouse anti-human 4F2hc (Chemicon, Temecula, CA, USA; 1 : 250 dilution) and mouse anti-human *α*-tubulin (Sigma, 1 : 10 000 dilution). After incubation for 1 h, membranes were washed and incubated with the secondary antibodies: horse radish peroxidase-linked anti-mouse (Jackson ImmunoResearch Laboratories Inc., West Grove, PA, USA; 1 : 10 000 dilution) or horse radish peroxidase-linked anti-rabbit IgG (Jackson ImmunoResearch Laboratories; 1 : 2000 dilution). After incubation for 1 h, Amersham (GE Healthcare, Piscataway, NJ, USA) ECL Plus™ western blotting detection reagents were used for visualisation.

### Immunofluorescence microscopy

Pancreatic cancer cells used for immunocytochemistry were cultured on glass chamber slides. Cells were washed two times in PBS and fixed in 4% paraformaldehyde for 10 min. Following three washes in PBS and 30 min incubation in blocking solution (PBS plus 5% goat serum plus 0.1% Triton X-100), cells were incubated with rabbit anti-mouse xCT antibody (Transgenic Inc., Kumamoto, Japan; 1 : 50 dilution) or mouse anti-human 4F2hc antibody (Chemicon; 1 : 200 dilution), diluted in blocking solution for 45 min at 4°C. Cells were then rinsed three times with blocking solution and incubated with either goat anti-mouse or goat anti-rabbit AlexaFluor®594 (Invitrogen; 1 : 100 dilution) for 30 min in the dark. Finally, cells were washed, stained with 4′6-diamidino-2-phenylindole (DAPI) and mounted with Vectashield® (Vector Laboratories, Burlingame, CA, USA). Immunofluorescence was detected with an Axiovert 40 imaging microscope (Carl Zeiss Canada, Toronto, ON, Canada), and images were captured with a AxioCam HRC digital camera (Carl Zeiss). Merged images were generated using Adobe PhotoShop (Adobe Systems, Mountain View, CA, USA).

Primary human pancreatic tumours and corresponding normal human pancreatic tissues (gift of Dr Sylvia Ng, BC Cancer Agency, Vancouver, BC, Canada.) were embedded and frozen in Tissue-Tek® optimal cutting temperature compound (Somagen Diagnostics, Edmonton, Alberta, Canada), sectioned at 5-*μ*m thickness using a CM1850 UV cryostat (Leica, Wetzlar, Germany), and mounted on Fisherbrand Plus microscope slides. Subsequently, these samples were subjected to the same immunohistochemical staining procedures as described for cultured cells. The patient samples were obtained in agreement with the Ethical Committee of the University of British Columbia and after obtaining informed consent from the patients.

### Transient transfection

Plasmid constructs of mouse xCT and 4F2hc cDNAs cloned into the pcDNA3.1^+^ vector were transformed into the *E.coli* strain DH5*α*, and plasmid DNA harvested using a Qiagen Maxiprep kit according to the manufacturer's recommendations.

For x_c_^−^ transporter overexpression studies, pancreatic cancer cells were plated at a density of 0.5 × 10^3^ cells per well in 96-well plates. After 24 h, wells were aspirated and 125 *μ*l of fresh media were added. Cells were transiently transfected with 0.1 *μ*g DNA (empty vector pcDNA3.1 control, pcDNA3.1-xCT, or pcDNA3.1-4F2hc) diluted in 12.5 *μ*l 150 mM NaCl and 0.75 *μ*l ExGen 500 *in vitro* transfection reagent (Fermentas, Burlington, ON, Canada) for 24 h.

### Glutamate uptake assay

Glutamate uptake by cultured cells was used to measure x_c_^−^ transporter activity as described previously (Shih and Murphy, 2001). Briefly, cells were plated in 24-well plates at 5 × 10^5^ cells per well and incubated overnight. Cells were washed with and pre-incubated in 1 ml per well Na^+^-free buffer A consisting of 140 mM *N*-methyl-D-glucamine, 5.4 mM KCl, 0.4 mM KH_2_PO_4_, 10 mM HEPES, 5 mM D-glucose, 1.8 mM CaCl_2_, 0.8 mM MgSO_4_ (pH 7.4) for 20 min at 37°C. The medium was then replaced with 300 *μ*l Na^+^-free buffer A containing 33 nM L-[^3^H]-glutamate (49 Ci mmol^−1^) (Amersham Pharmacia/GE Healthcare, Pittsburg, PA, USA) in the presence or absence of 1 *μ*M unlabeled amino-acid competitors (L-glutamate, L-cystine) or non-competitors (L-leucine) for 20 min at 37°C. Uptake was terminated by washing three times with ice-cold Na^+^-free buffer A, after which cells were solubilised with 200 *μ*l 0.5% Triton X-100 in 0.1 M potassium phosphate buffer (pH 7.0). To determine intracellular L-[^3^H]-glutamate uptake, 100 *μ*l of cell lysate was mixed with 5 ml of scintillation cocktail (Fisher, Pittsburg, PA, USA), and radioactivity was measured using an LKB Wallac 1214 Rackbeta (American Instrument Exchange Inc., Haverhill, MA, USA) liquid scintillation counter. A 10 *μ*l aliquot of cell lysate was used in a BCA protein assay kit (Pierce Chemical Co, Rockford, IL, USA) to determine protein concentration.

### Statistical analysis

Student's *t*-test was used unless otherwise stated to determine statistical significance. Results with a *P*⩽0.05 were considered significant.

## Results

### Growth requirement for extracellular cystine

**Figure 1:**
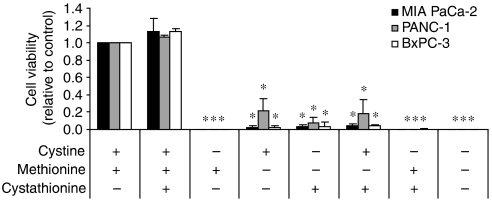
Pancreatic cancer cells depend on extracellular cystine for growth. Neutral red uptake assay for cell proliferation in MIA PaCa-2, PANC-1, and BxPC-3 cells incubated in medium in the presence or absence of methionine (0.1 mM), cystine (0.1 mM), and/or cystathionine (0.15 mM), in the combinations indicated for 72 h. Data represent the mean±s.e.m. from three independent experiments. ^*^P<0.01.

It is known that cysteine, the reduced form of cystine, is a non-essential amino acid in the human diet as tissues in the body such as the liver can generate cysteine through the transsulphuration pathway (Stabler *et al*, 1993; Brosnan and Brosnan, 2006). This pathway involves the metabolism of methionine, a nutritionally essential amino acid, to a cystathionine intermediate and its subsequent cleavage by *γ*-cystathionase to *α*-ketobutyrate and cysteine (Stabler *et al*, 1993; Brosnan and Brosnan, 2006). Certain cancer cells cannot generate their own cysteine/cystine and hence depend on extracellular sources to sustain growth and survival (Iglehart *et al*, 1977; Gout *et al*, 2001). To determine if pancreatic cancer cells require exogenous cystine for growth and survival, we cultured MIA PaCa-2, PANC-1, and BxPC-3 in the presence and absence of cystine, methionine, and/or cystathionine in all possible combinations. Survival and robust growth of all three cancer cell lines was observed only in cultures containing both methionine and cystine (Figure 1), demonstrating that the absence of either amino acid inhibited survival and proliferation *in vitro*. Cystathionine, which can substitute for cystine in some cell systems (Uren and Lazarus, 1979), failed to promote cell survival/growth when added to cystine-deficient, methionine-containing cultures (Figure 1). These results indicate that pancreatic cancer cell lines are dependent on uptake of cystine from their microenvironment for growth and survival, and suggest that the enzymes involved in the transsulphuration pathway may not be present or activated in these cells.

### A negative correlation exists between extracellular cystine deprivation and expression of the x_c_^−^ transporter

**Figure 2:**
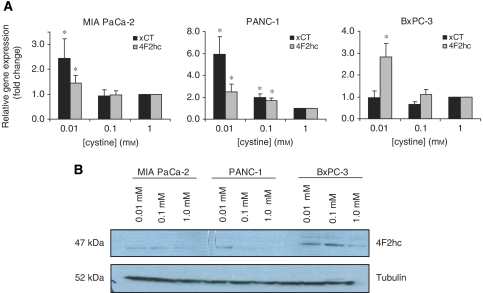
A negative correlation exists between extracellular cystine deprivation and expression of the x_c_^−^ transporter. (**A**) Q-RT–PCR for expression of xCT or 4F2hc mRNA in MIA PaCa-2, PANC-1, and BxPC-3 cell lines incubated with various cystine concentrations (0.01, 0.1 or 1 mM) for 72 h. Data represent the mean±s.e.m. from three independent experiments. ^*^P⩽0.05. (**B**) Western blot for expression of 4F2hc or tubulin in MIA PaCa-2, PANC-1, and BxPC-3 cell lines incubated with various cystine concentrations (0.01, 0.1 or 1 mM) for 72 h. Results are representative of two independent experiments.

The x_c_^−^ transporter is a major transporter of extracellular cystine (Bannai, 1984b). To determine whether extracellular cystine concentrations affect the expression of the x_c_^−^ transporter, pancreatic cancer cells were incubated in a medium containing low (0.01 mM), normal (0.1 mM) or high (1.0 mM) levels of cystine for up to 72 h. Cells in media containing low cystine exhibited signs of death after 72 h (data not shown). The x_c_^−^ transporter is structurally composed of an xCT light subunit, which confers substrate specificity and a 4F2hc heavy subunit, which is a common subunit of many amino-acid transporters (Sato *et al*, 1999; Bassi *et al*, 2001). The mRNA expression levels of the xCT and 4F2hc subunits at varying cystine concentrations were determined by quantitative real-time RT–PCR (q-RT–PCR). In response to low cystine concentrations, xCT mRNA was elevated in two of the three cell lines (MIA PaCa-2 and PANC-1), whereas 4F2hc mRNA was elevated in all three cell lines (MIA PaCa-2, PANC-1 and BxPC-3) (Figure 2A). Expression of 4F2hc protein in all three cell lines was determined by western blot analysis and yielded a similar increased expression level in response to low cystine concentration (Figure 2B). Unfortunately, no satisfactory xCT antibody for western blotting was available at the time these experiments were conducted, thus precluding the examination of xCT protein expression in our studies. These findings demonstrate that inverse correlations exist between extracellular cystine concentration and both xCT and 4F2hc expression in some pancreatic cancer cell lines, suggesting that these cells can modulate the expression of x_c_^−^ transporter to accommodate their growth needs.

### Oxidative stress increases x_c_^−^ transporter expression and GSH levels

**Figure 3:**
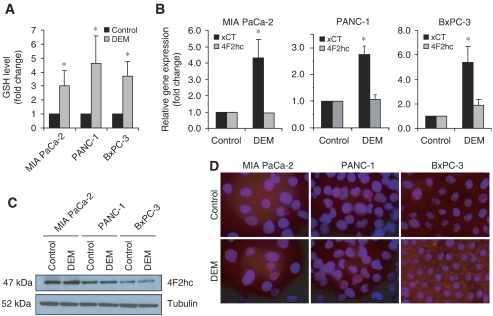
Oxidative stress increases x_c_^−^ transporter expression and GSH levels. (**A**) Intracellular GSH levels in MIA PaCa-2, PANC-1, and BxPC-3 cell lines either untreated or treated with 1 mM DEM for 24 h. Data represent the mean±s.e.m. from at least two independent experiments. ^*^P<0.05 using the Wilcoxon rank-sum test. (**B**) Q-RT–PCR for xCT and 4F2hc mRNA expression in MIA PaCa-2, PANC-1, and BxPC-3 cell lines either untreated or treated with 1 mM DEM for 24 h. Data represent the mean±s.e.m. from at least two independent experiments. ^*^P<0.05 (**C**) Western blot for expression of 4F2hc and tubulin in MIA PaCa-2, PANC-1, and BxPC-3 cell lines either untreated or treated with 1 mM DEM for 24 h. Results are representative of two independent experiments. (**D**) Immunofluorescence for xCT (red) and DAPI (blue) in MIA PaCa-2, PANC-1, and BxPC-3 cell lines either untreated or treated with 1 mM DEM for 24 h. Results are representative of two independent experiments.

The oxidative stressor, diethylmaleate (DEM), is commonly used to regulate intracellular GSH levels (Bannai, 1984a; Kim *et al*, 2001; Hosoya *et al*, 2002) and is often used to induce the expression of stress response-related genes. All three pancreatic cancer cell lines were treated with 1 mM DEM for 24 h, and an increase in total intracellular GSH levels in response to DEM treatment was confirmed (Figure 3A). In a blood–brain barrier cell line, treatment with DEM increased GSH levels with a corresponding increase in xCT mRNA expression (Hosoya *et al*, 2002). To determine whether the DEM-induced increase in GSH levels in pancreatic cancer cells corresponded with an increase in x_c_^−^ transporter expression, mRNA expression levels of xCT and 4F2hc were determined by q-RT–PCR. xCT mRNA expression was significantly upregulated in all three cell lines in response to DEM (Figure 3B). In contrast, 4F2hc mRNA remained at control levels (Figure 3B), consistent with previous studies that reported no effect of DEM on 4F2hc mRNA expression in a rat retinal capillary endothelial cell line (Tomi *et al*, 2002) and a human retinal pigment epithelial cell line (Bridges *et al*, 2001). Corresponding 4F2hc protein levels also remained unchanged in response to DEM treatment (Figure 3C). To assess xCT protein expression, immunofluorescent staining with an anti-xCT antibody was performed on pancreatic cancer cell lines. Increased xCT protein levels were observed in all three pancreatic cancer cell lines in response to DEM treatment, with the BxPC-3 cell line exhibiting a clear localisation of the xCT protein to the plasma membrane upon treatment with DEM (Figure 3D). These findings suggest that pancreatic cancer cells, in response to oxidative stress, upregulate expression of the x_c_^−^ transporter by inducing xCT (but not 4F2hc) subunit expression, resulting in a corresponding increase in GSH synthesis. This increase in GSH synthesis may in turn enable pancreatic cancer cells to survive in the presence of elevated levels of reactive oxygen species.

### Expression of the x_c_^−^ transporter in primary human pancreatic cancer specimens

**Figure 4:**
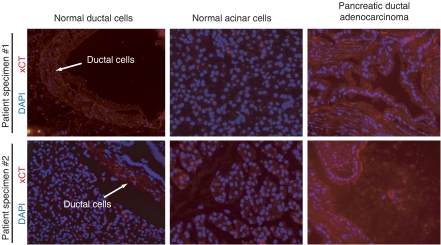
Expression of the x_c_^−^ transporter in primary human pancreatic cancer specimens. Immunofluorescence for xCT (red) and DAPI (blue) in normal ductal cells, normal acinar cells, and pancreatic ductal adenocarcinoma cells from two primary human pancreatic cancer patient specimens.

Having demonstrated that pancreatic cancer cell lines express the x_c_^−^ transporter, its expression was assessed in primary human pancreatic cancer tissues. Owing to difficulties in obtaining fresh human pancreatic cancer tissues, only two patient samples were used in this study. Both pancreatic cancer patient specimens with corresponding normal pancreatic tissue from the same patient were stained with anti-xCT antibody by immunofluorescence. Use of a rabbit IgG antibody as a negative control indicated that the xCT staining was not due to non-specific binding of immunoglobulins. In the normal pancreatic tissues, xCT protein expression was primarily localised to ductal cells, not acinar cells (Figure 4). Importantly, the pancreatic ductal adenocarcinomas exhibited overexpression of xCT protein. The distinct histological features of the two pancreatic cancer specimens, including atypical epithelial architecture, papillary folding, and intraluminal cell shedding, indicated that these specimens were likely invasive pancreatic ductal adenocarcinomas. The upregulated expression of xCT protein in the pancreatic cancer tissues suggest that elevated x_c_^−^ transporter expression plays a role in the pathogenesis of pancreatic cancer.

### A positive correlation exists between the expression level of xCT and resistance towards GEM

**Figure 5:**
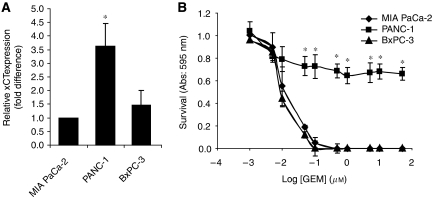
A positive correlation exists between the expression level of xCT and resistance towards GEM. (**A**) Q-RT–PCR for xCT expression in MIA PaCa-2, PANC-1, and BxPC-3 cell lines. Data represent the mean±s.e.m. from three independent experiments. ^*^P<0.05. (**B**) Neutral red uptake assay for cell proliferation in Mia PaCa-2, PANC-1, and BxPC-3 cultures treated with increasing concentrations of GEM for 72 h. Data represent the mean±s.e.m. from three independent experiments. ^*^P<0.01.

Thus far we have obtained evidence that elevated x_c_^−^ transporter expression can enhance pancreatic cancer cell growth by promoting the uptake of extracellular cystine, which in turn functions to maintain high levels of intracellular GSH to promote cancer cell survival in the presence of oxidative stress. Among the three pancreatic cancer cell lines used in this study, PANC-1 cells expressed the highest relative level of xCT mRNA compared with that of BxPC-3 and MIA PaCa-2 (Figure 5A), suggesting that PANC-1 cells may exhibit the greatest resistance towards oxidative stress-induced cell death. Gemcitabine, the most common chemotherapeutic agent for the treatment of pancreatic cancer (Maehara *et al*, 2004), induces cell death through a mechanism that can involve oxidative stress (Maehara *et al*, 2004; Donadelli *et al*, 2007). To determine whether a relationship exists between the expression level of the x_c_^−^ transporter and resistance towards GEM, we treated pancreatic cancer cells with GEM and determined the half maximal inhibitory concentration (IC_50_) of GEM using neutral red assays. The IC_50_ of GEM was highest in PANC-1 cells (>50 *μ*M) compared with MIA PaCa-2 (∼0.03 *μ*M) and BxPC-3 (0.01 *μ*M) cells (Figure 5B). The higher resistance of the PANC-1 cells to GEM may correspond with the higher number of cystine transporters per cell as suggested by relative xCT expression levels. Hence a negative correlation exists between the expression level of xCT and sensitivity to GEM; PANC-1 cells, which have highest relative expression of xCT, are the least sensitive to GEM, whereas MIA PaCa-2 cells, which have the lowest relative expression of xCT, are the most sensitive to GEM.

### Overexpression of transfected xCT increases cystine uptake and increases GEM resistance

**Figure 6:**
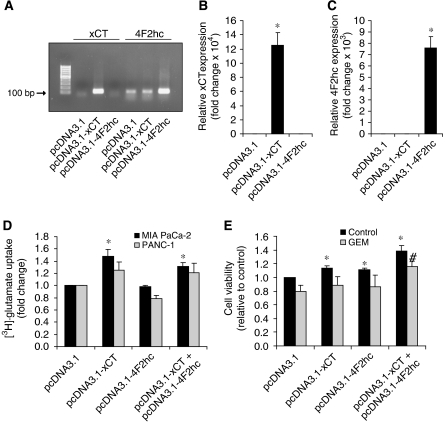
Overexpression of transfected xCT increases cystine uptake and increases GEM resistance. (**A**) RT–PCR for xCT and 4F2hc expression in MIA PaCa-2 cells transfected with pcDNA3.1 empty vector, pcDNA3.1-xCT plasmid, or pcDNA3.1-4F2hc plasmid. (**B**, **C**) Q-RT–PCR for xCT (**B**) or 4F2hc (**C**) expression in MIA PaCa-2 cells transfected with pcDNA3.1 empty vector, pcDNA3.1-xCT plasmid, or pcDNA3.1-4F2hc plasmid. ^*^P<0.001. (**D**) [^3^H]-glutamate uptake assay in MIA PaCa-2 and PANC-1 cells transfected with pcDNA3.1 empty vector, pcDNA3.1-xCT plasmid, and/or pcDNA3.1-4F2hc plasmid. ^*^P<0.01 compared with respective cell line pcDNA3.1 control. (**E**) Neutral red uptake assay for survival of untreated or GEM-treated MIA PaCa-2 cells transfected with pcDNA3.1 empty vector, pcDNA3.1-xCT plasmid, and/or pcDNA3.1-4F2hc plasmid. ^*^P⩽0.05 compared with pcDNA3.1 control; ^#^*P*⩽0.05 compared with pcDNA3.1 GEM treatment. All data represent the mean±s.e.m. from three independent experiments.

To determine if overexpression of the x_c_^−^ transporter can increase resistance to GEM, xCT and 4F2hc cDNAs were transfected into the pancreatic cell lines MIA PaCa-2 and PANC-1. Increased xCT and 4F2hc mRNA expression was confirmed by semi-quantitative RT–PCR and q-RT–PCR in transfected MIA PaCa-2 (Figure 6A–C) and PANC-1 (data not shown) cell lines. Using a radioactive glutamate uptake assay, MIA PaCa-2 cells transfected with either xCT cDNA alone or both xCT and 4F2hc cDNA exhibited increased x_c_^−^ transport activity (Figure 6D). Transfection of 4F2hc cDNA alone, however, was not sufficient to upregulate x_c_^−^ transporter activity (Figure 6D). Furthermore, transfection of both xCT and 4F2hc cDNA was required to increase GEM resistance in MIA PaCa-2 cells (Figure 6E). Hence upregulation of the xCT subunit expression is necessary to upregulate x_c_^−^ transporter activity, with a corresponding increase in GEM resistance. These results suggest that overexpression of the x_c_^−^ transporter, as seen in primary pancreatic ductal adenocarcinoma specimens, may increase pancreatic cancer cell survival in response to GEM treatment.

### Targeting x_c_^−^ transporter function with monosodium glutamate

**Figure 7:**
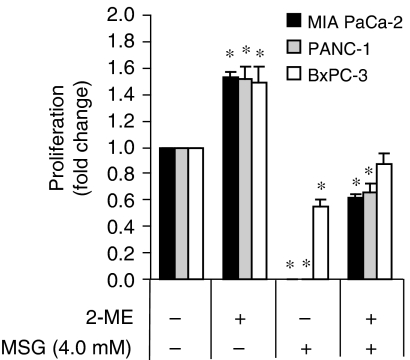
Targeting x_c_^−^ transporter function with MSG. Neutral red uptake assay for cell proliferation in MIA PaCa-2, PANC-1, and BxPC-3 cultures treated with MSG (4.0 mM) or 2-ME (66 *μ*M) in various combinations for 72 h. ^*^P⩽0.05. All data represent the mean±s.e.m. from three independent experiments.

Monosodium glutamate (MSG) is a specific inhibitor of the x_c_^−^ transporter as the uptake of cystine can be competitively inhibited by glutamate (Bannai, 1986). Consistent with this, pancreatic cancer cells treated with MSG (4.0 mM) exhibited markedly inhibited growth, with growth of MIA PaCa-2 and PANC-1 cultures completely abrogated. Importantly, the growth arrest could be largely prevented by including 2-mercaptoethanol (2-ME; 66 *μ*M) in the culture medium, a compound commonly used to shuttle cystine into cells through the L-transport system hence circumventing inhibition of the x_c_^−^ transporter (Ishii *et al*, 1981) (Figure 7). Taken together, our findings demonstrate that the growth of pancreatic cancer cells can be inhibited by specific inhibition of the x_c_^−^ transporter.

## Discussion

The x_c_^−^ transporter has been shown in various cancers to supply cells with cystine for GSH synthesis resulting in increased proliferation and GSH-related drug resistance (Doxsee *et al*, 2007; Narang *et al*, 2007). The goal of this study was to investigate whether a similar x_c_^−^-mediated system was also used by pancreatic cancer cells for growth, survival and drug-related resistance.

In the present study, the human pancreatic cancer cell lines MIA PaCa-2, PANC-1, and BxPC-3 were determined to be critically dependent on extracellular cystine for growth (Figure 1). This finding demonstrates that the biochemical cysteine synthesis pathway known as the transsulphuration pathway does not participate in alleviating cystine depletion in the cell lines of this study. Some cell types, such as neurons and astrocytes, do not rely on the x_c_^−^ transporter for growth (Chung *et al*, 2005). As such, these cells may (i) possess a functional transsulphuration pathway, (ii) express other transporters that mediate the transport of cystine or cysteine into the cell, for example, excitatory amino-acid transporters (McBean and Flynn, 2001), or (iii) not express the x_c_^−^ transporter, but instead rely solely on uptake of extracellular cysteine secreted by certain somatic cells (Gout *et al*, 2001; Chung *et al*, 2005; Lo *et al*, 2008). The potential utility of screening tumour biopsies for the presence of enzymes in the transsulphuration pathway (i.e., for *γ*-cystathionase) to determine whether a patient may benefit from cystine starvation therapy remains a possibility.

Because our results indicate that pancreatic cancer cell lines are dependent on extracellular cystine/cysteine for growth, these cell lines may exhibit sensitivity towards depletion of the amino acid in their environment. Indeed, at least two of the three cell lines tested showed an inverse correlation between a low extracellular cystine environment (0.01 mM) and expression of the x_c_^−^ transporter, suggesting that these cells can modulate their expression of the x_c_^−^ transporter to accommodate their growth needs. Consistent with published reports, cell proliferation is strongly associated with cystine/cysteine availability and intracellular GSH levels (Godwin *et al*, 1992; Noda *et al*, 2002).

Besides manipulating cysteine/cystine levels in the extracellular microenvironment to effect a change in x_c_^−^ transporter expression, the addition of the oxidative stressor DEM was also used. In accordance with other studies (Bannai, 1984a; Kim *et al*, 2001; Hosoya *et al*, 2002), DEM treatment induced xCT mRNA expression with a corresponding increase in total GSH, indicating that increased intracellular GSH synthesis enables pancreatic cancer cells to survive in the presence of oxidative stress. In human embryonic kidney (HEK) cells, xCT expression has been reported at the plasma membrane (Shih and Murphy, 2001). Of interest is the BxPC-3 cell line, which upon treatment with DEM, clearly exhibited localisation of the xCT subunit to the plasma membrane (Figure 3D). The 4F2hc subunit is known to translocate other light subunits to the plasma membrane for functional activity to occur (Chillaron *et al*, 2001; Verrey *et al*, 2004), suggesting that xCT subunits located in the plasma membrane of BxPC-3 cells are likely functional. The fact that prominent xCT protein localisation to the plasma membrane was not seen in the other cell lines tested may be explained by the observation that BxPC-3 cells morphologically grow in tighter groups of cells, facilitating the visualisation of xCT protein at cell–cell (intercellular) plasma membrane junctions. In contrast to BxPC-3 cells, DEM induced upregulation of xCT protein in MIA PaCa-2 and PANC-1 cells within the intracellular compartment (Figure 3D). Indeed, subcellular localisation studies of xCT in HEK cells have reported xCT expression not only at the plasma membrane, but also at intracellular membranes such as the lysosomal or endosomal membranes (Shih and Murphy, 2001). The functional significance of intracellular xCT expression, however, remains to be determined.

Although use of the oxidative stressor DEM was able to increase xCT expression, it did not have an effect on 4F2hc mRNA or protein expression (Figure 3B and C). The xCT promoter region contains an antioxidant response element (ARE) that regulates transcription of the gene (Wasserman and Fahl, 1997; Ishii *et al*, 2000). In contrast, the presence of an ARE has not been reported in the 4F2hc promoter region, suggesting a possible explanation for the observed difference in response to oxidative stress for the two genes. Alternatively, basal levels of 4F2hc may be much higher than those of xCT, potentially rendering changes in 4F2hc expression undetectable. Indeed, in a blood–retinal barrier cell line, mRNA levels of 4F2hc were reported to be 56-fold higher than those of xCT (Tomi *et al*, 2002). This suggests that 4F2hc mRNA in some cells is in excess, most likely because of the fact that 4F2hc is a common component of many other amino-acid transport systems.

Among normal tissues, xCT is expressed predominantly in normal pancreas (Bassi *et al*, 2001; Kim *et al*, 2001), specifically in the islet cell population (Bassi *et al*, 2001). Furthermore, xCT has also been reported to exhibit higher expression in an acinar cell line compared with a pancreatic islet cell line (Sato *et al*, 1998). The present study shows that xCT is expressed in normal pancreatic tissues preferentially in the ductal cells compared with the acinar cells. Importantly, in primary human pancreatic ductal adenocarcinoma specimens, xCT protein is overexpressed throughout the cancerous ductal structures (Figure 4). Given that the x_c_^−^ transporter is overexpressed in pancreatic cancers, and that expression of the x_c_^−^ transporter can be induced in pancreatic cancer cell lines in response to oxidative stress, our findings implicate the x_c_^−^ transporter as an important mediator of pancreatic cancer cell proliferation and survival.

The higher xCT expression by the PANC-1 cells suggest that they have more x_c_^−^ cystine transporters per cell than the other cells and hence a greater ability to take up cystine from the microenvironment, likely synthesising more GSH when needed. It has been reported that NIH3T3 cells treated with sublethal levels of DEM to induce ROS defense genes such as xCT and glutathione-*S*-transferase *α*1 (GST*α*1) are associated with higher resistance to various apoptotic stimuli such as buthionine sulphoximine, ultraviolet, and etoposide (Faraonio *et al*, 2006). Moreover, HT22 hippocampal cells resistant to oxidative stress exhibited a 7-fold overexpression of xCT mRNA, with a concomitant increase in resistance to glutamate-induced oxidative stress and hydrogen peroxide-induced oxidative stress (Lewerenz *et al*, 2006). In the present study, although PANC-1 cells express a 3.5-fold higher level of xCT mRNA compared with the MIA PaCa-2 and BxPC-3 cell lines, PANC-1 cells are ∼1000 × more resistant to GEM. This suggests that GEM resistance cannot be solely explained by higher xCT mRNA expression, but rather that additional GEM resistance mechanisms are at play. Nonetheless, overexpressing xCT and 4F2hc in pancreatic cancer cells resulted in an overall increase in the GEM resistant phenotype (Figure 6). The fact that overexpressing the x_c_^−^ transporter was able to increase cell survival even in the absence of GEM treatment highlights the importance of the x_c_^−^ transporter as a regulator of pancreatic cancer cell proliferation and survival. Furthermore MSG, a potent and specific inhibitor of the x_c_^−^ transporter (Bannai, 1986), significantly inhibited the growth of the pancreatic cancer cell lines. By including 2-ME (66 *μ*M) in the culture medium, which provides an alternative route for cystine uptake through L-transport system (Ishii *et al*, 1981), growth arrest was prevented. This gives direct evidence that the pancreatic cancer cell lines critically depend on the x_c_^−^ transporter for growth and survival. Unfortunately, MSG cannot be used for therapy as it is neurotoxic (Choi, 1988).

Our results demonstrate that pancreatic cancer cells exhibit upregulated expression of the x_c_^−^ transporter, which promotes uptake of extracellular cystine for synthesis of GSH. In turn, elevated intracellular GSH levels promote pancreatic cancer cell growth and survival in response to the production of reactive oxygen species by oxidative stressors, including the chemotherapeutic agent GEM. Hence, drug resistance in pancreatic cancer may be explained in part by overexpression of the x_c_^−^ transporter. Finally, the marked growth arrest of the pancreatic cancer cell lines obtained through specific inhibition of the x_c_^−^ transporter suggests that this transporter may serve as a target for therapy of the disease.
